# Beyond Trauma: Splenic Rupture While Surfing Revealing Occult Infectious Mononucleosis

**DOI:** 10.7759/cureus.117560

**Published:** 2026-10-06

**Authors:** Letícia Heeren, Adelaide Gomes da Costa, Regina Silva, Eva Carvalho, Agata Ferreira

**Affiliations:** 1 General Surgery, Unidade Local de Saúde do Oeste, Caldas da Rainha, PRT; 2 General Surgery, Centro Hospitalar do Oeste, Caldas da Rainha, PRT

**Keywords:** atraumatic splenic injury, epstein-barr virus, haemoperitoneum, infectious mononucleosis, splenectomy, splenic rupture, splenomegaly

## Abstract

When a young patient collapses with haemoperitoneum after physical activity, trauma is the presumptive diagnosis; Epstein-Barr virus (EBV) infectious mononucleosis (IM) is easily overlooked. However, IM carries a rare but potentially fatal complication: splenic rupture, occurring in approximately 0.1-0.5% of symptomatic cases. We report the case of a previously healthy 21-year-old man who presented with sudden-onset abdominal pain and presyncope while surfing, without any fall or direct abdominal impact. Over the subsequent two hours, he developed progressive haemodynamic instability. A focused assessment with sonography for trauma (FAST) was positive for free intraperitoneal fluid. Following initial resuscitation, contrast-enhanced computed tomography demonstrated a grade V splenic injury, splenomegaly, and massive haemoperitoneum, prompting emergency splenectomy. Intraoperatively, the spleen was markedly enlarged, approximately three times the expected normal weight, friable, and extensively lacerated. The initial diagnosis was traumatic splenic rupture. However, on postoperative day three, persistent fever prompted investigation for an infectious cause. Serology confirmed acute EBV infection. This case highlights that severe splenic rupture occurring during ordinary physical activity, particularly when accompanied by splenomegaly disproportionate to the reported mechanism, should prompt postoperative evaluation for EBV infection, even in the absence of classic symptoms of IM. Earlier recognition of the underlying diagnosis might also help avoid unnecessary investigations and treatments during the postoperative period.

## Introduction

Infectious mononucleosis (IM), caused by Epstein-Barr virus (EBV, human herpesvirus 4), is one of the most prevalent viral infections of adolescents and young adults, classically presenting with fever, pharyngotonsillitis, and lymphadenopathy. Splenic involvement is nearly universal during acute infection, with lymphocytic infiltration and follicular hyperplasia producing splenomegaly in up to 75% of symptomatic patients [1,2]. Spontaneous splenic rupture represents the most severe and life-threatening complication, occurring in approximately 0.1-0.5% of symptomatic cases [1,3].

A systematic review of 186 published cases found a clear male predominance, with rupture most often occurring within three weeks of symptom onset [1]. Only 9.1% of cases had an antecedent traumatic event documented; the remaining cases occurred spontaneously or, as in the present case, during ordinary physical activity without identifiable injury. The pathophysiological basis is a spleen structurally compromised by rapid capsular distension, follicular expansion, and vascular congestion, an organ susceptible to rupture from forces, including exertion, that would not threaten an anatomically normal spleen [1,3,4].

The diagnostic challenge is greatest when the diagnosis of IM has not been established at the time of rupture, and when an apparently plausible mechanical explanation exists. In this situation, the infectious aetiology is easily overlooked, and the patient is managed as a primary trauma case. Recognition of the true diagnosis may then depend entirely on incidental intraoperative findings or the subsequent postoperative clinical course [5-7].

We report a case of grade V splenic rupture in a previously healthy young man without prodromal symptoms, in whom the diagnosis of EBV infectious mononucleosis was established only after splenectomy. We discuss the diagnostic clues that emerged during the patient's clinical course and the practical implications for postoperative management.

## Case presentation

A previously healthy 21-year-old man with no relevant medical history presented to the emergency department after developing acute left-sided abdominal pain and dizziness during recreational surfing. He denied any fall, collision, or direct impact while surfing. He described a near-syncopal episode immediately after leaving the water and sustained a witnessed syncopal episode in the emergency department waiting room shortly after arrival. He denied any preceding fever, sore throat, odynophagia, or constitutional symptoms in the preceding days or weeks.

On initial assessment, vital signs were as follows: blood pressure of 113/59 mmHg, heart rate of 60 bpm, and peripheral oxygen saturation of 99% on room air, with sinus rhythm on ECG. Initial laboratory investigations showed haemoglobin of 8.8 g/dL, leucocytosis of 11,700/µL, and elevated transaminases - aspartate aminotransferase (AST) of 120 U/L, alanine transaminase (ALT) of 273 U/L, and alkaline phosphatase (ALP) of 168 U/L - with C-reactive protein (CRP) of 0.6 mg/L.

Progressive haemodynamic deterioration was documented: blood pressure fell to 93/35 mmHg, with a heart rate of 83 bpm. Arterial blood gas showed a pH of 7.43, pO₂ of 94 mmHg, and lactate of 1.0 mmol/L. A focused assessment with sonography for trauma (FAST) was positive for free intra-abdominal fluid. Intravenous fluid resuscitation with 1,000 mL isotonic saline was initiated, and a contrast-enhanced CT was performed following fluid-mediated stabilisation.

Contrast-enhanced CT of the abdomen and pelvis demonstrated a splenic rupture with a markedly enlarged spleen measuring 15 cm in maximum dimension, an extensive subcapsular haematoma, and signs of active haemorrhage from the splenic parenchyma into the peritoneal cavity, with a moderate-to-large haemoperitoneum. The findings were classified as an American Association for the Surgery of Trauma (AAST) 2018 grade V splenic injury [8], and the patient was taken immediately to the operating room for emergency laparotomy (Figures 1-4).

**Figure 1:**
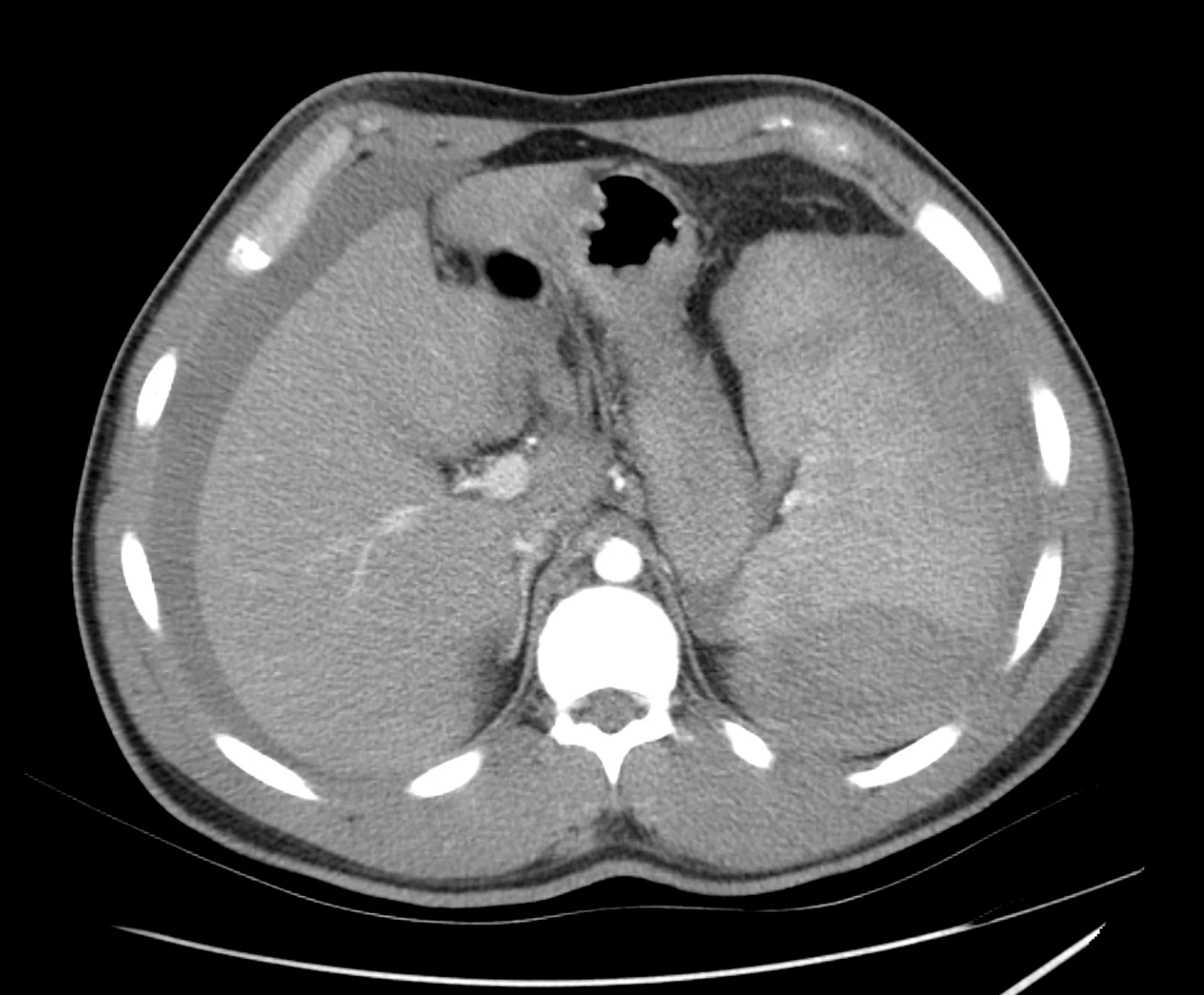
Axial contrast-enhanced CT image at the level of the spleen, demonstrating a hypodense area within the splenic parenchyma consistent with laceration/haematoma

**Figure 2:**
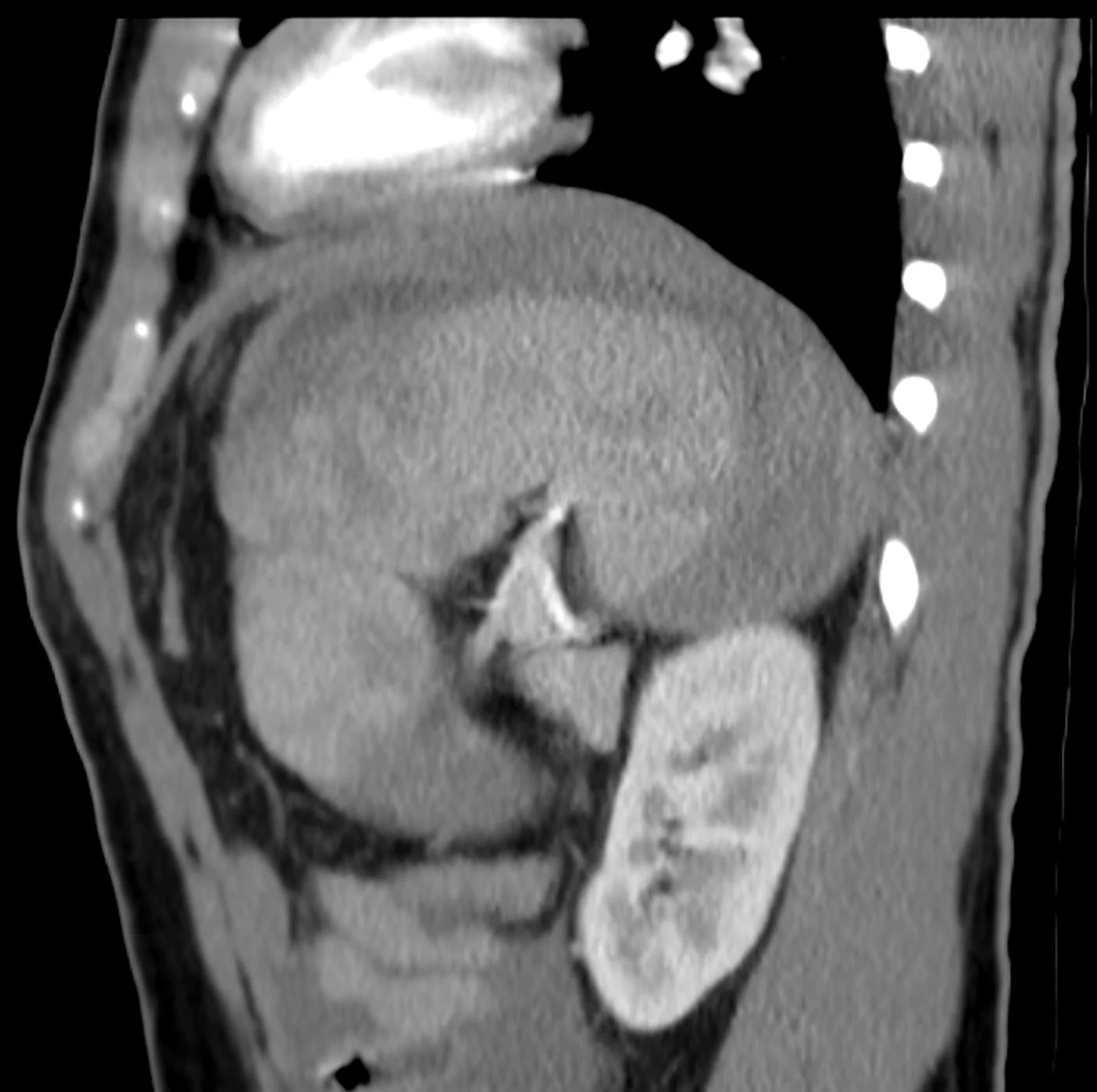
Sagittal CT image showing marked splenomegaly with heterogeneous parenchymal attenuation

**Figure 3:**
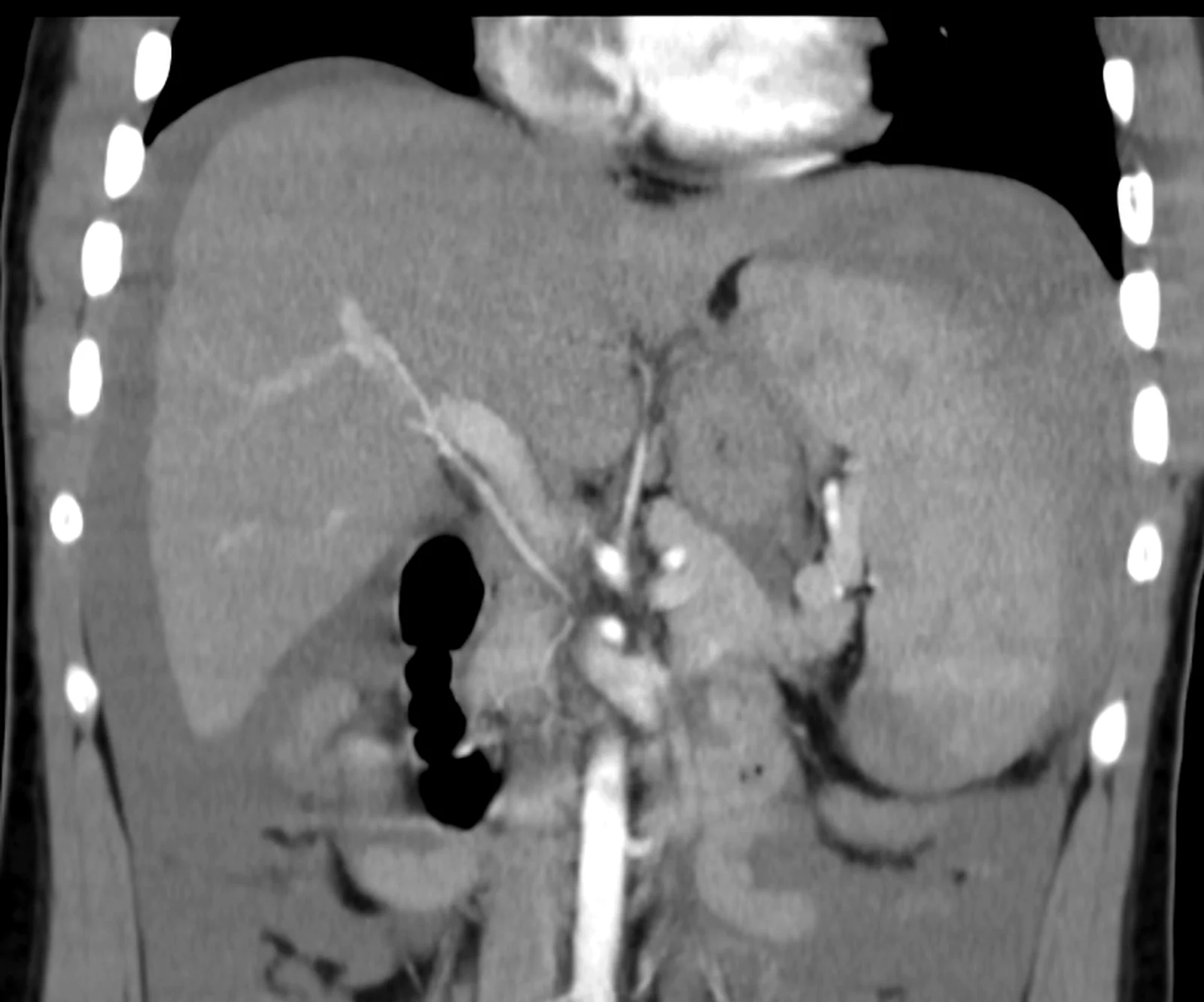
Coronal CT image demonstrating splenic enlargement relative to the liver

**Figure 4:**
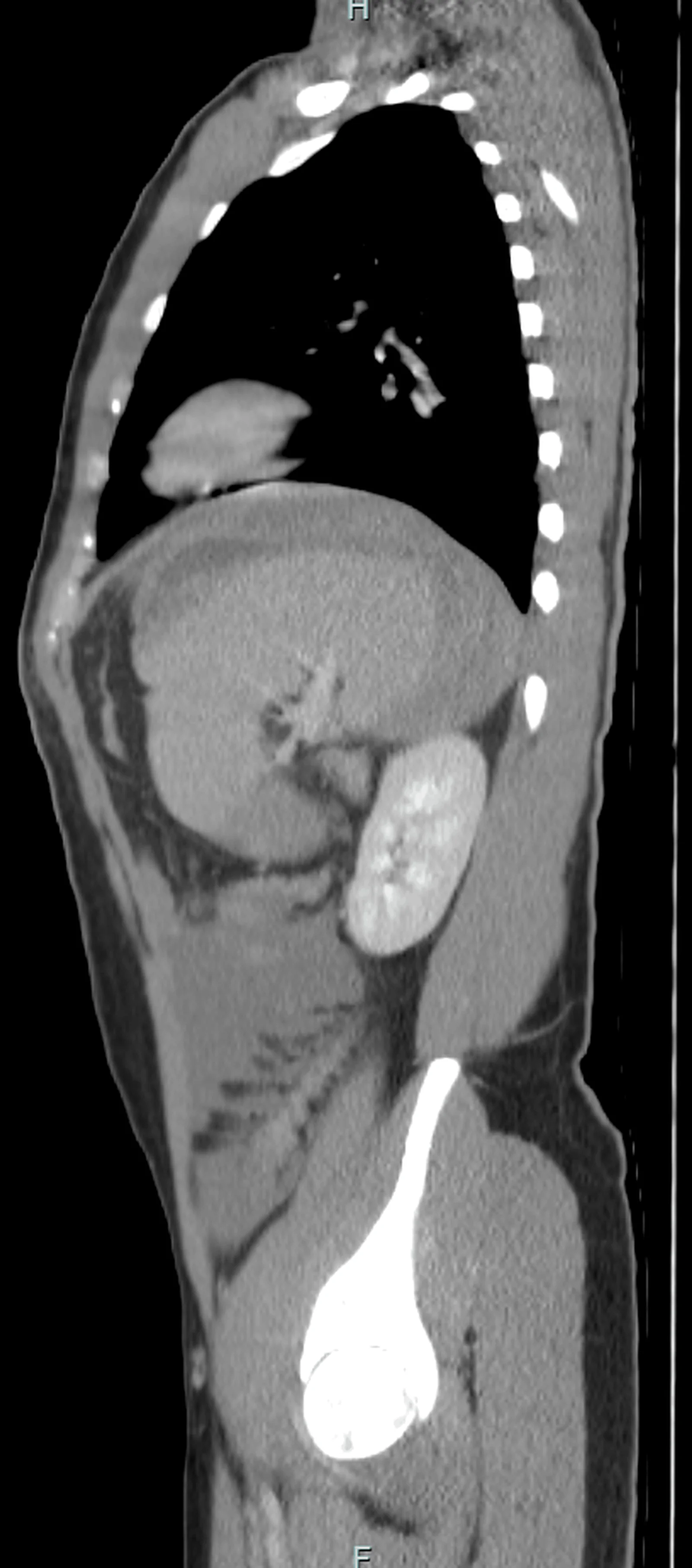
Whole-body sagittal CT reconstruction demonstrating the enlarged spleen with adjacent perisplenic haemoperitoneum extending into the pelvis

Upon entering the abdominal cavity, approximately 2 L of haemoperitoneum were evacuated. The spleen was found to be massively enlarged, far exceeding what would be expected from the reported mechanism, friable, and extensively disrupted, with the capsule almost entirely absent and multiple parenchymal lacerations. The marked splenomegaly and near-complete loss of capsular integrity made splenectomy technically challenging (Figure 5). Splenic preservation was not feasible. A splenectomy was performed. Two units of packed red blood cells (pRBCs) were transfused intra-operatively; haemodynamics remained stable throughout the procedure. The resected specimen was sent for histopathological examination.

**Figure 5:**
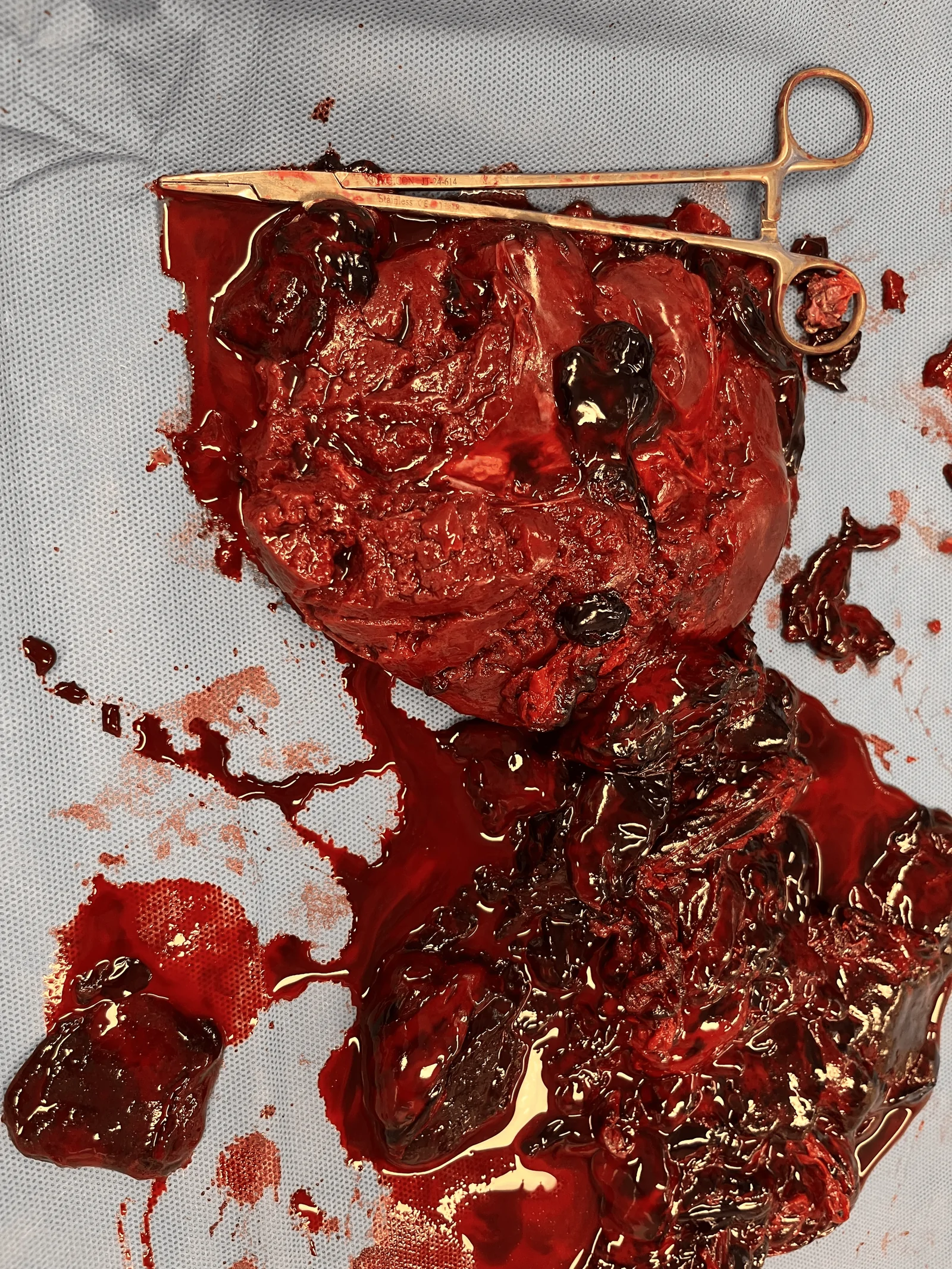
Gross intraoperative appearance of the resected spleen The specimen is markedly enlarged with extensive capsular disruption, multiple haemorrhagic parenchymal lacerations, and loss of normal splenic architecture, consistent with a grade V injury.

The spleen weighed 407 g, approximately three times the expected normal weight and measured 17×13.7×15.7 cm. Macroscopically, the capsule was almost entirely absent, with extensive parenchymal lacerations, haemorrhagic infiltration, and intraparenchymal haematomas. The pathological conclusion was traumatic splenic lesions, with no other alterations of pathological significance identified. No specific histological features of lymphoid hyperplasia or EBV-associated changes were reported, likely due to the extensive haemorrhagic disruption of the normal splenic architecture.

Postoperative course and diagnosis

Haemoglobin was 8.3 g/dL on postoperative day (POD) 1 and decreased to 7.9 g/dL on POD 2, prompting transfusion of one additional unit of packed red blood cells. By POD 3, it had increased to 10.1 g/dL. That same day, the patient developed fever. Given the concern for an early postoperative complication, a contrast-enhanced abdominal CT scan was performed, which showed no evidence of intra-abdominal collections, bleeding, or other surgical complications. On POD 4, the patient remained febrile, and he complained of a sore throat. Examination of the oropharynx revealed bilateral exudative tonsillitis with whitish plaques. The combination of postoperative fever, the pharyngeal findings, and the retrospective recognition of the marked splenomegaly already identified on preoperative imaging and confirmed at surgery raised suspicion of an underlying infectious process. Serological testing for EBV, together with a broad infectious work-up, was therefore requested.

Blood tests on POD 4 showed a leukocyte count of 22,200/µL, with lymphocyte predominance (60%), thrombocytosis (580,000/µL), worsening of transaminase levels (ALT of 457 U/L, AST of 180 U/L, GGT of 179 U/L, and LDH of 346 U/L), and a CRP level of 2.2 mg/L (Table 1). Autoimmune hepatitis was excluded (negative antinuclear antibody (ANA), antimitochondrial antibody (AMA), and anti-liver-kidney microsomal type 1 (anti-LKM1) antibodies), and cytomegalovirus serology was negative. EBV serology demonstrated positive viral capsid antigen (VCA) IgM and IgG together with Epstein-Barr nuclear antigen (EBNA) IgG, consistent with a recent EBV infection (Table 2).

**Table 1:** Trend of haematological and biochemical parameters during admission and the postoperative course ALP: alkaline phosphatase; ALT: alanine aminotransferase; AST: aspartate aminotransferase; CRP: C-reactive protein; GGT: gamma-glutamyl transferase; LDH: lactate dehydrogenase; POD: postoperative day

Parameter	Admission	POD 1	POD 2	POD 3	POD 4	Reference range
Haemoglobin	8.8	8.3	7.9	10.1	-	13.5-17.5 (g/dL)
Leukocytes	11,700	14,900	14,300	-	22,200	4,000-11,000 (/µL)
Lymphocytes	52.1	50.1	47.5	-	60	20-40 (%)
Platelets	168,000	223,000	301,000	-	580,000	150,000-450,000 (/µL)
AST	120	-	-	-	180	10-40 (U/L)
ALT	273	-	-	-	457	7-56 (U/L)
ALP	168	-	-	-	-	44-147 (U/L)
GGT	-	-	-	-	179	8-61 (U/L)
LDH	-	-	-	-	346	140-280 (U/L)
CRP	0.6	-	-	-	2.2	<5 (mg/L)

**Table 2:** Serological and autoimmune work-up performed on POD 4 AMA: anti-mitochondrial antibody; ANA: anti-nuclear antibody; CMV: cytomegalovirus; EBNA: Epstein-Barr nuclear antigen; EBV: Epstein-Barr virus; IgG: immunoglobulin G; IgM: immunoglobulin M; LKM1: liver-kidney microsomal antibody type 1; VCA: viral capsid antigen

Test	Result	Interpretation
ANA	Negative	Autoimmune hepatitis excluded
AMA	Negative	Primary biliary cholangitis/autoimmune liver disease excluded
Anti-LKM1	Negative	Autoimmune hepatitis excluded
CMV serology	Negative	CMV infection excluded
EBV VCA IgM	Positive	Marker of acute/recent EBV infection
EBV VCA IgG	Positive	Consistent with EBV infection
EBV EBNA IgG	Positive	Atypical timing; discussed under serological pattern

Amoxicillin-clavulanate was prescribed empirically for one week after the diagnosis of exudative tonsillitis, before the serological results became available. No throat swab was obtained to exclude streptococcal pharyngitis. Notably, the patient did not develop the maculopapular rash classically associated with aminopenicillin administration during EBV infectious mononucleosis. Its absence here illustrates that a lack of this classic sign should not be taken to exclude an underlying EBV process. The patient's clinical condition subsequently improved, and he was discharged on POD 8.

## Discussion

This case raises three questions that we believe are of practical relevance to emergency physicians and surgeons: why did a grade V splenic rupture occur during ordinary physical activity without any trauma? What should have directed us to investigate EBV earlier? What would have been done differently if the diagnosis had been made sooner?

The initial and entirely natural assumption on presentation was that this was a traumatic splenic injury sustained during surfing. The patient himself reported no significant impact, which was unexplained at the time, but was attributed to the possibility that a minor, unrecognised blow had occurred. The marked splenomegaly already identified on contrast-enhanced CT was the first finding to challenge this assumption, and the disproportionate size and friability of the organ were subsequently confirmed intraoperatively.

This finding reframes the entire clinical narrative. EBV infection produces intense lymphocytic infiltration and follicular hyperplasia within the spleen, leading to rapid expansion of the organ and thinning of its capsule [1,3,4]. The result is a spleen that can rupture under mechanical forces insufficient to injure a normal organ: during a fall, a cough, or, as in this case, the ordinary physical exertion of a recreational activity [9-11]. In the largest published series, only 9.1% of EBV-associated splenic ruptures were preceded by identifiable trauma [1] - meaning that, in the great majority of cases, as in this case, the rupture occurs during routine activity or at rest.

The histopathological report is instructive in this context. Despite a spleen weighing 407 g and measuring 17×13.7×15.7 cm, the pathologist identified no specific features of lymphoid hyperplasia or EBV-associated changes. This does not contradict the serologically confirmed diagnosis; rather, it reflects the destructive extent of a grade V injury, in which haemorrhagic obliteration of the normal splenic architecture renders histological identification of EBV changes unreliable. Surgeons and pathologists should be aware that the absence of specific histological features does not exclude an underlying infectious aetiology in this context.

Looking back, there were signals at admission that deserved more attention. The transaminase elevation on the first blood panel (ALT of 273 U/L, AST of 120 U/L) was not trivial. While minor enzyme elevation can accompany haemorrhagic shock, this degree of elevation is not a typical feature of isolated traumatic haemoperitoneum. EBV hepatitis occurs in the majority of patients with IM and can precede other clinical features [2]; in retrospect, this early biochemical finding was an overlooked marker of underlying viral hepatic involvement. Had the significance of this elevation been recognised at admission, particularly in the context of a young male patient with haemoperitoneum and no clear traumatic mechanism, earlier infectious investigation might have been prompted, even if it would not have changed the surgical decision-making given the haemodynamic instability.

What ultimately pointed the clinical team toward EBV was not biochemistry alone, but the combination of persistent fever and the development of exudative tonsillitis on POD 4. Importantly, the haematological and biochemical abnormalities were already present before surgery. Lymphocytosis was evident on admission (52.1% of 11,700/µL leukocytes), increasing to 60% of 22,200/µL leukocytes on POD 4, corresponding to an absolute lymphocyte count of approximately 13,300/µL. The pre-existing elevation of transaminases, with a subsequent increase in AST from 120 U/L to 180 U/L and ALT from 273 U/L to 457 U/L by POD 4, was also compatible with EBV-associated hepatic involvement. In contrast, the marked postoperative thrombocytosis (580,000/µL) is more appropriately interpreted as a reactive consequence of splenectomy rather than as a specific manifestation of EBV. Taken together, the preoperative lymphocytosis and transaminitis, subsequent marked lymphocytosis, persistent fever and exudative tonsillitis, together with the massive splenomegaly identified on imaging and confirmed intraoperatively, provided a coherent clinical picture suggestive of acute EBV infection.

This sequence illustrates an important practical lesson: when splenomegaly appears disproportionate to the apparent mechanism of splenic injury, it should prompt consideration of an underlying systemic process. In the presence of lymphocytosis, transaminitis and compatible clinical features, EBV infection should be included among the differential diagnoses and investigated appropriately.

What earlier diagnosis would have changed

A specific management decision in the postoperative period would have been avoided had the diagnosis of IM been established earlier. The first was the empirical prescription of amoxicillin-clavulanate for the exudative tonsillitis. This was initiated before serological results were available, without throat culture, in a setting of diagnostic uncertainty between bacterial pharyngitis and viral tonsillitis. In the context of confirmed EBV infection, antibiotic therapy is not indicated for the pharyngotonsillitis and carries a well-described risk of a drug-induced maculopapular rash in patients with IM [2]. The second was the repeat CT scan performed on POD 3 in response to fever - a reasonable decision given the need to exclude an operative complication, but one that exposed the patient to additional radiation and procedural burden. Had the fever and tonsillitis been contextualised earlier within a diagnosis of EBV infection, the threshold for reimaging might reasonably have been higher. These two points are not presented as errors of management - the decisions were clinically justified given the information available at the time - but as concrete illustrations of how earlier recognition of the underlying diagnosis translates into fewer unnecessary interventions.

The serological pattern

The concurrent positivity of VCA IgM, VCA IgG, and EBNA IgG is worth addressing, as it departs from the classic pattern of primary EBV infection in which EBNA IgG typically appears weeks to months after acute disease onset. In the present case, the positive EBNA IgG could suggest either reactivation of a prior infection - though VCA IgM positivity is atypical in reactivation - or early EBNA seroconversion, which has been described in immunocompetent adults; the latter would be compatible with a late primary infection or early seroconversion phase, suggesting that the infection may have preceded the splenic rupture by several weeks rather than representing an incidental finding. The positive VCA IgM remains the cornerstone serological marker of acute or recent EBV disease, and in the context of the full clinical picture - splenomegaly, hepatitis, lymphocytosis, tonsillitis, and negative alternative investigations - the diagnosis of active EBV infection is strongly supported. Clinicians should nonetheless be aware that non-classical serological profiles occur and, in cases of diagnostic uncertainty, infectious disease specialist input should be sought [2]. A limitation of the diagnostic work-up performed in this case is that hepatic synthetic function (INR, bilirubin, and albumin) and viral hepatitis A, B, and C serologies were not assessed, since the transaminase elevation was, at the time, attributed to the acute surgical presentation rather than to a separate hepatic process; these would have been informative additions to more fully characterise the hepatic involvement described.

## Conclusions

Splenic rupture complicating EBV infectious mononucleosis can occur during ordinary physical activity, without any identifiable traumatic event, and may be the first and only clinical manifestation of an otherwise unsuspected underlying infection. The extent of splenic injury and the intraoperative findings may themselves provide the first diagnostic clues. When a surgeon encounters splenomegaly disproportionate to the reported mechanism of injury, this should prompt consideration of EBV infection and other relevant underlying causes, even when the presentation initially appears traumatic.

In this case, earlier recognition of the EBV aetiology would have prevented the empirical antibiotic treatment of a viral tonsillitis, and a repeat CT scan performed to exclude an operative complication that was in fact an infectious manifestation. Admission transaminase elevation, though non-specific, was a retrospectively visible early clue. These observations reinforce the value of maintaining a broad differential diagnosis in young patients with haemoperitoneum and no compelling traumatic mechanism.
